# Managing Corneal Infections: Out with the old, in with the new?

**DOI:** 10.3390/antibiotics12081334

**Published:** 2023-08-18

**Authors:** Sanjay Marasini, Jennifer P. Craig, Simon J. Dean, Leon G. Leanse

**Affiliations:** 1 Department of Ophthalmology, New Zealand National Eye Centre, The University of Auckland, Auckland 1142, New Zealand; s.marasini@auckland.ac.nz (S.M.); jp.craig@auckland.ac.nz (J.P.C.); simondean@xtra.co.nz (S.J.D.); 2 Health and Sports Sciences Hub, Europa Point Campus, University of Gibraltar, Gibraltar GX11 1AA, Gibraltar; 3 Wellman Center for Photomedicine, Massachusetts General Hospital, Harvard Medical School, Boston, MA 02114, USA

**Keywords:** antimicrobial blue light, corneal collagen crosslinking, corneal ulcer, eye infections, microbial keratitis, phage therapy, probiotics, ultraviolet C light

## Abstract

There have been multiple reports of eye infections caused by antibiotic-resistant bacteria, with increasing evidence of ineffective treatment outcomes from existing therapies. With respect to corneal infections, the most commonly used antibiotics (fluoroquinolones, aminoglycosides, and cephalosporines) are demonstrating reduced efficacy against bacterial keratitis isolates. While traditional methods are losing efficacy, several novel technologies are under investigation, including light-based anti-infective technology with or without chemical substrates, phage therapy, and probiotics. Many of these methods show non-selective antimicrobial activity with potential development as broad-spectrum antimicrobial agents. Multiple preclinical studies and a limited number of clinical case studies have confirmed the efficacy of some of these novel methods. However, given the rapid evolution of corneal infections, their treatment requires rapid institution to limit the impact on vision and prevent complications such as scarring and corneal perforation. Given their rapid effects on microbial viability, light-based technologies seem particularly promising in this regard.

## 1. The Eye in Health and Disease

The cornea is the major refractive medium of the visual system, possessing complex and highly ordered optical characteristics [1]. The smooth wet surface of the ocular surface is maintained by the tear film, a complex amalgamation of lipids, proteins, mucins, defensins, and electrolytes [1,2]. The ocular surface harbors multiple genera of potentially pathogenic bacteria, including (but not limited to) *Pseudomonas* spp., *Propionibacterium* spp., *Corynebacterium* spp., *Acinetobacter* spp., *Staphylococci* spp., and *Streptococcus* spp. [3]. Despite the abundance and diversity of such pathogens, a healthy ocular surface resists microbial invasion. The defense from infections is passively provided by several anatomical (e.g., eyelids and eyelashes), physical (epithelial intercellular tight junctions and regular desquamation), and chemical barriers (e.g., proteins in the tear film such as lysozyme, defensins, and lactoferrin, which serve to inhibit bacterial growth, adherence, and survival) [4]. When an infection overwhelms the ‘passive’ defense mechanism, the ‘active’ defense mechanism switches on, leading to acute inflammation that eradicates specific pathogens (e.g., pattern-recognition receptors that detect unique pathogen-associated molecular patterns (PAMPs); toll-like receptors that recognize bacteria, viruses, fungi and protozoa; and cytokines, chemokines, and effector cells that initiate and amplify the immune response to clear the pathogens) [4,5]. The compromise in active and passive defense mechanisms predisposes the ocular surface to various infiltrative events and infections [6].

## 2. Ocular Infections and Therapeutic Challenges

All types of pathogens (e.g., bacteria, fungi, viruses, and protozoans) can invade the eye, and disease management is based on targeted treatment towards causative microorganisms. In 2012, 77 species from 42 genera of bacteria alone were identified to be associated with corneal ulcers [7]. This diversity of organisms contributes to the need for targeted treatment [8]. However, the drugs that historically were effective against microorganisms several decades ago are demonstrating an ongoing loss of efficacy. This issue applies to all drug classes, including antibiotics, antifungals and antiviral drugs, and infection types such as infectious conjunctivitis [9], corneal infections [8], and endophthalmitis [10].

**Figure 1:**
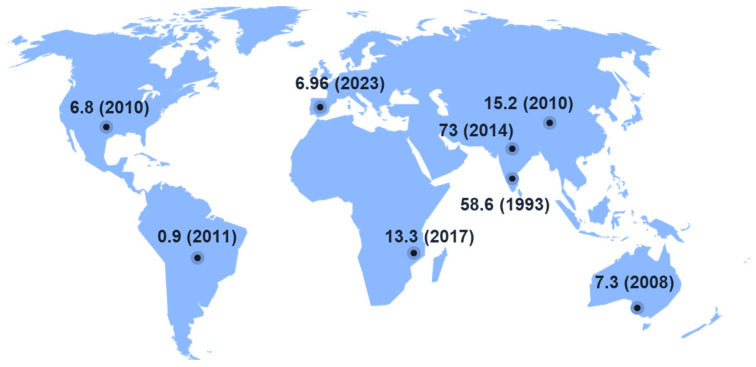
Incidence of microbial keratitis per 100,000 people.

The disease incidence and antibiotic resistance pattern in eye infections varies across continents and countries. For example, the reported incidence of culture-proven microbial keratitis (MK) in Scotland was approximately 0.26 per 10,000 individuals [11], with higher rates of up to 4.2 per 10,000 observed in contact lens wearers [12]. In Nottingham, UK, the estimated incidence of culture-proven MK was 3.47 per 10,000 individuals [13]. Similarly, the reported rates are as high as 11.3 per 10,000 individuals in India [14] and 79.9 per 10,000 individuals in Nepal [15] (Figure 1). The annual incidence of MK has been continually rising worldwide, attributed to a changing pattern of predisposing factors [16]. For example, the increase in the use of contact lenses worldwide [17] has been linked to an increase in infections caused by *P. aeruginosa* [18,19]. Gram-positive isolates account for most cases of bacterial keratitis worldwide, and *Staphylococcal* species are the most common [20,21,22]. Among Gram-negative bacteria, incidence rates vary, especially for *Pseudomonas* species. [23]. The proportion of *Moraxella* species in bacterial keratitis is reported to have decreased, while that of *Pseudomonas* and *Serratia* species has increased since the late 1990s [24].

Active lesions in viral keratitis caused by herpes simplex and herpes zoster virus generally respond favorably to antiviral drugs (e.g., acyclovir), although more advanced infections such as geographic ulcers react slowly and indolent ulcers may show no response to treatment at all [25]. There have been reports of acyclovir-resistant herpes simplex virus keratitis [26], and overall resistance among immunocompromised patients has increased to 6.4% [27,28]. Resistance to acyclovir occurs because of viral thymidylate kinase and DNA polymerase mutations that decrease this enzyme’s affinity for its substrate [29]. Fungal keratitis is treated using antifungal agents. Antifungal agents are divided into three main groups: polyenes (such as amphotericin B, natamycin, and nystatin); azoles (ketoconazole, miconazole, econazole, fluconazole, itraconazole, voriconazole, and posaconazole); and allylamine (terbinafine) and echinocandins (caspofungin), with natamycin being the only antifungal ophthalmic solution that is commercially available globally [30]. Fungal keratitis is a sight-threatening infection and can lead to severe visual loss and even loss of the eye. Although conventional antifungal agents are generally successful in fungal keratitis, there have been multiple reports of fungal keratitis showing resistance to these drugs [31,32]. Acanthamoeba keratitis affects immunocompetent healthy individuals and is usually treated using dual therapy with polyhexamethylene biguanide (PHMB) and hexamidine [33]. Generally, the treatment works well if initiated early in the infection course. However, there have been reports of drug-resistant Acanthamoeba isolates [34].

Generally, the risk factors for superficial eye infections are well defined and include contact lens wear (21.7% to 50.3%) [20,35], trauma (15.0–24.0%) [35,36], ocular surface disease (18.0% to 21.0%) [20,35], corneal surgery (4.0% to 30.0%) [20,35,36], or systemic disease [37,38]. The causative microorganism can generally be predicted based on the risk factor and associated signs and symptoms—for example, *Acanthamoeba* infection is mostly associated with contact lens wear [39]. A laboratory culture report confirms the diagnosis, and a negative culture often complicates disease management, therefore, empiric treatment is often valuable. However, in most cases, a treatment is best tailored to individual needs. When a bacterial species is suspected, some clinicians prefer a combination therapy consisting of a cephalosporin (e.g., cefuroxime 5.0%) and aminoglycoside (e.g., tobramycin 1.35%) to cover a wide range of both Gram-negative and Gram-positive bacteria (dual therapy) [40], while others prefer empirical fluoroquinolone monotherapy [41], until a culture report is available. Aminoglycosides show strong bactericidal activity, especially against Gram-negative bacteria, by inhibiting bacterial protein synthesis [42]. Cephalosporins are more effective against Gram-positive bacteria by inhibiting cell wall synthesis via interference with peptidoglycan [43]. Therefore, a combination treatment tends to be highly effective against bacterial keratitis. Fluoroquinolones are synthetic broad-spectrum antibiotics which inhibit the DNA gyrase and topoisomerase enzymes, which are the key enzymes involved in in DNA replication and transcription. Inhibition of these enzymes leads to cell death [44]. In Australia, between 2001 and 2003, 95.0% of all prescriptions for bacterial keratitis were for fluoroquinolone monotherapy, of which 80.0% were ciprofloxacin [38]. Similar patterns of antibiotic prescriptions have been reported in recent years. In contrast, dual therapy was a preferred treatment method in Auckland, New Zealand, contributing to 64.0% of all prescriptions during the same period [45].

The initial (empirical) treatment choice depends on several factors, including disease severity, geography, risk factors, and the physician’s preference [46]. Also, a more recent study suggests that changing susceptibility patterns of keratitis isolates has also changed the practice patterns of corneal specialists across the world. The Cornea Society listserv conducted an online survey among its members, who were corneal specialists, to assess practice patterns in the initial treatment of bacterial ulcers [47]. It was observed that fortified vancomycin was more commonly prescribed by US practitioners relative to international practitioners (55% vs. 33%). Overall, 80% of US practitioners reported that they chose fortified antibiotics and 16% used a fourth-generation fluoroquinolone as empiric treatment. International respondents were twice as likely to use fluoroquinolone monotherapy (31%) and were less likely to use fortified vancomycin (33%). This is in line with the absence of vancomycin resistance and correspondingly low minimum inhibitory concentrations of ocular surface isolates in the US, as published in 2022 by the Antibiotic Resistance Monitoring in Ocular micRoorganisms (ARMOR) surveillance programs, which report antibiotic resistance rates and trends [48].

Although dual therapy demonstrates efficacy in treating bacterial keratitis (BK), many avoid its use as an initial therapy for reasons related to ocular toxicity and issues of compliance, as alternating eye drops typically need to be applied every half an hour [41]. Also, during the four years from 2001 to 2004, an increasing resistance of bacterial keratitis isolates to aminoglycosides was reported in China, primarily to tobramycin, at a rate of up to 29.0% in 2008 [23]. Similarly, ciprofloxacin has also demonstrated a loss of efficacy, with reported resistance rates of between 1% and 36% [49,50,51,52,53]. In India, bacterial resistance to ciprofloxacin has been reported to be 30.7% [54], and to have risen from 11.0% in 1990 to 28.0% in 1998 in South Florida [55], and from 5.8% in 1993 to 35.0% in 1997 in Pittsburgh, USA [53]. Although these reports highlight the growing inefficacy of ciprofloxacin against primarily Gram-positive cocci, higher resistance rates for ciprofloxacin have also previously been reported against Gram-negative bacilli [23]. Similarly, 5.0% of BK isolates resisted cefuroxime in Waikato, New Zealand, [56] and 33.3% resisted cefuroxime in Auckland, New Zealand, as reported within the last decade [8]. In Oxford, UK, 49.1% of BK isolates were reported to resist cefuroxime [57]. In Auckland, New Zealand, the resistance rates to penicillin, amoxicillin, and chloramphenicol were reported to be 57.1%, 41.6%, and 5.3%, respectively, over the 2013–14 period [8].

New fluoroquinolones have been developed to tackle antibiotic resistance issues in recent years. In China, 36.0% of BK isolates resisted ciprofloxacin, while only 15.5% resisted levofloxacin, a newer generation of fluoroquinolone [23]. Studies show similar treatment outcomes for infectious keratitis with moxifloxacin, a fourth-generation fluoroquinolone, relative to ciprofloxacin, or ofloxacin as well as to combined fortified cefazoline and tobramycin [58]. Although studies cannot be directly compared due to methodological and chronological differences, it is clear that antibiotics, which were considered a major line of defense against microorganisms only a few decades ago, are becoming increasingly limited in their effectiveness in treating today’s infections [59].

## 3. Ocular Infections: Overcoming Therapeutic Challenges with Novel Treatments

The rate of development of new antimicrobial agents has not matched the rate of increase in antimicrobial resistance. Only two new classes of antibiotics were introduced into the clinic between 1968 and 2003, neither of which was significantly effective against Gram-negative bacteria [60], although in 2017, a new class of drug, teixobactin, which thus far claims to be resistance-free, was approved by the FDA [61]. This drug inhibits cell wall synthesis by binding to a highly conserved motif of lipid II (precursor of peptidoglycan) and lipid III (precursor of cell wall teichoic acid). While the issue of antibiotic resistance and the gap in the development of new antibiotics is large, capital investment in antimicrobial research and development is small; less than 5% of total venture capital investment in pharmaceutical research and development was for antibiotic development between 2003 and 2013 [62,63]. For treating bacterial species that have been classified by the US Center for Disease Control and Prevention (CDC) as antibiotic-resistant bacteria that require urgent action, only five products were in the pipeline, with most in the early stages of development and none in clinical use, as reported in 2013. As of December 2018, four compounds in Phase II clinical trials represent novel classes of antibiotics, but these are considered insufficient to combat multidrug-resistant Gram-negative pathogens [64]. This is compounded by an historically high attrition rate for compounds making it through clinical trials to clinical utility. Approximately 90% of even drugs deemed safe and effective during preclinical research fail to translate to clinical utility, further complicating the drug development process [65].

Antibiotic resistance ranks among the top 10 causes of death in the United States, posing a substantial economic burden worldwide. In the US alone, annual costs associated with the loss of antibiotic effectiveness in outpatient prescriptions were estimated to be as high as USD 225 million [66]. The burden on the health-care system will likely continue to increase because the bacterial resistance rate is anticipated to continue to rise in the coming years. As a result, alternative approaches to controlling bacterial infections are needed. Within ophthalmology, due to compromised treatment efficacy as a result of antimicrobial resistance [67], it is now of the utmost importance to identify innovative strategies that extend the boundaries of current therapeutic platforms [68]. This issue is particularly emphasized by the concern arising from recent reports of contamination of ‘artificial tears’ by multidrug-resistant *Pseudomonas aeruginosa* [69]. In the next section, novel treatment approaches for ocular infections are discussed, focusing on microbial keratitis (MK). Specifically, the ways in which light-based anti-infective technology (e.g., photodynamic therapy, blue light, and ultraviolet C light), phage therapy, and probiotic approaches are attempting to shift the clinical paradigm to facilitate the treatment of antimicrobial-resistant ocular infections are discussed.

### 3.1. Light-Based Treatment Methods for Microbial Keratitis

Light is a known natural stressor to microorganisms, the use of which as a multifaceted therapeutic agent was proposed for the first time by Niels Ryberg Finson over a century ago. For this innovation, he was awarded a Nobel Prize in 1903 [70]. Recently, many studies have begun utilizing light to treat many diseases, including cancer, infectious diseases, and inflammatory diseases. As such, many studies have explored visible light or non-visible light as a method of eliminating ocular infections.

#### 3.1.1. Antimicrobial Photodynamic Therapy

**Figure 2:**
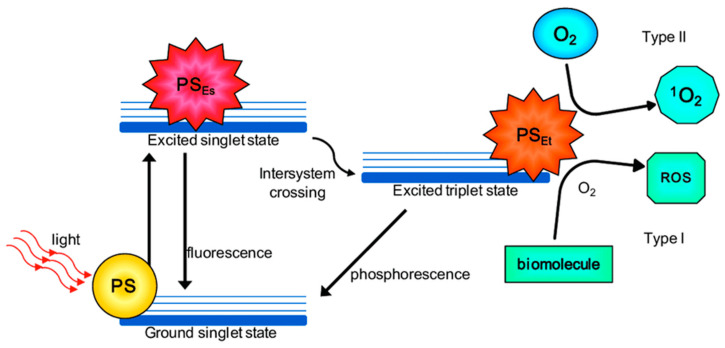
The types of photochemical reactions (type I and type II photochemistry) that can occur during photodynamic therapy. Reprinted with permission from ref. [74] 2016, MDPI.

Photodynamic therapy (PDT) is a light-based treatment approach that uses light in conjunction with a chemical photosensitizer in the presence of oxygen [62]. The result is induction of type I or type II photochemical reactions that generate reactive oxygen species (ROS), which include free radicals (e.g., hydroxyl radical, OH; superoxide anion, O_2_^•−^), molecules (e.g., hydrogen peroxide, H_2_O_2_; hypochlorous acid, HClO), and highly reactive singlet oxygen (^1^O_2_) (Figure 2). The type I reaction results in either hydrogen atom or electron transfer, yielding radicals or radical ions (e.g., superoxide and hydroxyl radicals), and the type II reaction leads mainly to singlet molecular oxygen by intersystem crossing [71]. These radicals are responsible for causing localized damage in a cell. Antioxidants (physical quenchers) rapidly remove ROS; therefore, ROS are short-lived and are restricted to a small subcellular volume surrounding the site of their production [72]. The effect is therefore localized to the target tissue. Antimicrobial PDT (aPDT) has been investigated for several decades and has shown promise for treating a myriad of infectious diseases. It has the advantage that it can rapidly eradicate microbes (within minutes), somewhat selectively, with a reduced likelihood of developing resistance relative to antibiotics [73]. Many photosensitizers that have been used are non-toxic and already within the clinical pipeline [62], suggesting potential for their use as an alternative antimicrobial strategy.

**Figure 3:**
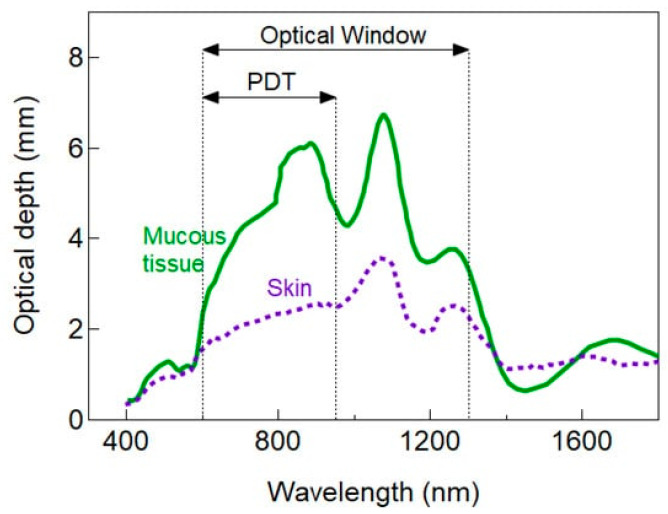
PDT optical window for skin and mucous tissue. Reprinted with permission from ref. [75] 2021, MDPI.

The light wavelength has a strong influence on its penetration into tissue. Therefore, there have been attempts to explore a favorable combination of wavelength and photosensitizers to optimize depth penetration, minimize potential toxicity of the chemical substrate, and maximize antimicrobial efficacy. In conventional PDT, longer wavelength visible light around the 660 nm spectral regions (i.e., red light) is frequently employed, resulting in a penetration depth that is significantly greater than shorter wavelength visible light (e.g., blue or green spectral regions). Employing longer wavelengths, coupled with a non-toxic photosensitizer, may therefore be a viable approach for the treatment of localized infections. Studies have estimated the PDT optical window for skin and mucosal tissues (Figure 3), for ophthalmic use; however, further investigation is still required. Nevertheless, given that the penetration of UVA (commonly employed in combination with riboflavin to treat microbial keratitis; see below) is significantly lower and is potentially more toxic than visible light at 660 nm, it may be reasonable to shift the paradigm slightly to consider ‘longer wavelength’ PDT as an option to treat ophthalmic infections.

#### 3.1.2. Ultraviolet A plus Riboflavin

Antimicrobial PDT has been applied clinically to treat MK in the form of ultraviolet A (UVA at 370 ± 5 nm wavelength) in combination with the photosensitizer, riboflavin, as corneal collagen crosslinking (CXL) [76]. This ‘crosslinking’ process produces more robust chemical bonds between adjacent fibrils, increasing their resilience against collagenase-induced degradation. While the underlying mechanism is not known precisely, it is understood to be driven by photooxidation that occurs both aerobically and anaerobically. During aerobic interactions, UVA induces riboflavin excitation, a triplet state reactive to molecular oxygen in a ground state. This process induces the production of ROS in singlet oxygen form through a type II photochemical reaction. Once the oxygen has been consumed during this reaction, the photochemistry switches towards a type I reaction, where the riboflavin begins to interact directly with the proteins present within the corneal stroma to produce ROS. The ROS by-products are responsible for the resulting ‘crosslink formation’ that occurs as they react further with the collagen and stromal proteins, ultimately enhancing corneal rigidity [77]. These ROS are also lethal to several microorganisms, including bacteria, fungi, Acanthamoeba, and viruses, as well as host cells. This antimicrobial property of CXL is sometimes used to manage resistant corneal infections [62]. Although CXL shows efficacy in managing resistant corneal ulcers caused by bacteria, fungi, and Acanthamoeba, outcomes from clinical trials are inconsistent. Additionally, available randomized clinical trials have shown a high risk of performance bias, further limiting their reliability [76]. The procedure has limitations due to its length, requiring between 30 and 60 min, along with the frequent application of riboflavin during light exposure and the requirement for corneal de-epithelization to improve chemical penetration. Attempts to reduce the treatment duration to as little as 2.5 min while exhibiting similar efficacies as the original protocol [78] suggest promise but further studies are required to confirm these findings. Long-term effects of corneal collagen crosslinking due to UVA exposure are not yet known but would be an important research question. Evidence of ocular herpes simplex virus reactivation with an increased UV index outdoors presents a theoretical risk that UVA exposure in CXL may aggravate viral corneal infections [79] or a latent viral disease, such as stromal keratitis. Therefore, important research questions and gaps in knowledge remain concerning the use of CXL technology for managing corneal infections.

#### 3.1.3. Red Light (660 nm) plus Methylene Blue

Studies have explored the use of aPDT methods using long-wavelength visible light. Unlike corneal collagen crosslinking, these methods are not yet clinically approved for patient use. A red light (660 nm) and methylene blue [7-bis(dimethylamino)-phenothiazin-5-ium chloride dye] photodynamic reaction (MB-PDT) has been observed to eliminate numerous infectious agents, including those associated with eye infections [80,81]. In a study, the MB-PDT technique was tested *in vitro* and *in vivo* for its potential to treat *Mycobacterium fortuitum* keratitis, an infection caused by a fast-growing non-tuberculous bacterium following ophthalmic surgery [82]. It was found that no detectable bacteria were observed after a radiant exposure of 100 J/cm^2^ of red light in combination with 0.01% MB in *in vitro* experiments. When MB-PDT was combined with amikacin eye drops, antimicrobial efficacy increased. The synergistic MB-PDT and amikacin treatment also proved highly effective in managing *M. fortuitum* rabbit corneal infections. In searching for individual candidate chemicals to use as a chemical substrate in aPDT, methylene blue is thus considered effective, and a combination therapy with antibiotics seems to enhance efficacy.

#### 3.1.4. Red Light plus Toluidine Blue O

Red light with peak absorption at 633 nm has been studied for its use in aPDT using Toluidine Blue O (TBO) as a chemical substrate. TBO is an acidophilic metachromatic dye that has high affinity for nucleic acids and polysaccharides. As a cost-effective chemical substrate, this dye has been studied for its potential photosensitizing effects to eliminate infectious agents [83] and has even been explored as a potential anticancer treatment [84]. TBO absorbs red light (peak absorption at 633 nm) and, upon excitation, undergoes a type II photochemical reaction generating singlet oxygen [85]. TBO-mediated photoactivated chromophores for infectious keratitis (PACT-CXL) have previously been shown to have antibacterial efficacy *in vitro* on *S. epidermidis* and *S. aureus* isolated from ocular surface infection, demonstrating its potential use as a chemical substrate in such infections [86]. TBO at concentrations ranging from 20 to 80 μM in combination with red light (625 nm) for 20 min (7.3 mW/cm^2^) demonstrated extensive bacterial killing. For *S. aureus* and *S. epidermidis*, 90% and 95% reductions in bacteria were noted, respectively, following red light exposure with 80 μM Toluidine Blue O [86]. Although TBO showed high antimicrobial efficacy against ocular pathogens, its safety to the ocular surface needs to be explored before being considered for clinical application.

#### 3.1.5. Red Light plus Chlorin e6

The application of Chlorin e6 as a photosensitizer for excitation with both red light and blue light for the treatment of pathogenic microbes has been examined by numerous studies [87,88]. It has additionally been tested as a photosensitizer and is clinically approved for treating different cancers. Chlorin e6 is a second-generation chlorin-based photosensitizer structurally comparable to porphyrin [89]. Upon photon excitation via intersystem crossing, ground-state molecular oxygen is converted into reactive singlet oxygen, which can kill cancerous cells or microbes. Using Chlorin e6, aPDT has been tested against multidrug-resistant *Staphylococcus aureus*, a common etiological agent of refractory corneal infection [90]. The aPDT was evaluated against 12 isolates of multidrug-resistant *Staphylococcus aureus*. The exposure resulted in a 5-log_10_ CFU reduction in bacterial viability when exposed to approximately 128 μM of Chlorin e6 combined with 18.6 J/cm^2^ red light (670 nm). In a staphyloxanthin (powerful antioxidant present in *S. aureus*)-deficient mutant of *S. aureus*, the susceptibility to aPDT was significantly greater than that of the wild-type *S. aureus*, an observation that has been similarly made in other light-based investigations [91]. Therefore, with further validation, red light might also prove valuable in aPDT for treating corneal infections caused by drug-resistant bacteria. However, an important consideration regarding this study is that it was performed *in vitro*. Thus, the clinical potential of using Chlorin-e6 against ophthalmic infections, both from an efficacy and safety perspective (especially given the potent oxidative potential of the photosensitizer), requires further study.

#### 3.1.6. Green Light plus Rose Bengal

Green light with a peak absorption of 500–551 nm has been studied for its efficacy in aPDT along with the chemical substrate rose bengal (4,5,6,7-tetrachloro 2′,4′,5′,7′-tetraiodo). Rose bengal is a fluorescein derivative that absorbs light within the green spectral region (550 nm peak absorption) [92] and has been tested as a photosensitizer to eliminate cancer cells and for its anti-infective properties against numerous infectious agents [93,94,95], including those responsible for ocular infections [96,97,98]. Like many photosensitizers, it shows an affinity to Gram-positive bacteria, likely due to the lack of outer membrane that is characteristic of Gram-negative bacteria. Halili et al. investigated rose bengal and riboflavin-mediated aPDT to treat *methicillin-resistant Staphylococcus aureus* (MRSA) [98]. Their study found that 5.4 J/cm^2^ of green light (500–551 nm; 30 min) combined with 0.1% or 0.03% rose bengal could completely inhibit MRSA. Therefore, a combination of green light and rose bengal is considered a potential aPDT technology for keratitis treatment. This study provides promising preliminary evidence for eliminating ocular pathogens, using MRSA as a representative etiological agent. Given that a multitude of different bacterial species (and other microbial species) may be implicated in the establishment of ocular infections, it is conceivable that this approach may not be equally effective for ocular infections of Gram-negative bacterial origin (e.g., *P. aeruginosa*), due to the more impermeable outer membrane. More recently, Sepulveda-Beltran retrospectively evaluated the clinical outcomes of rose bengal photodynamic reaction in infectious keratitis in a clinical sample of 31 eyes [99]. They observed clinical resolution in 77.4% of patients, with 22.5% requiring therapeutic penetrating keratoplasty and 54.8% requiring optical penetrating keratoplasty. A mix of pathogens was noted as the cause of infection, including 51.6% *Acanthamoeba* spp., 12.9% *Fusarium* spp., and 6.5% *Pseudomonas* spp. Despite limited research in clinical populations with this technique [99,100], results seem encouraging for managing corneal infections caused by a wide range of pathogens and further research is needed to consolidate these findings.

#### 3.1.7. Ultraviolet C Light for the Treatment of Ocular Infection

Ultraviolet C (200–280 nm), or UVC, is a well-established antimicrobial agent receiving interest as a treatment for surface or topical wound infections [101,102]. Its therapeutic potential for treating corneal infection has been demonstrated in preclinical studies [76,103,104]. UVC (265 ± 5 nm) is highly microbicidal as it is rapidly absorbed by the nucleic acids in the DNA, causing photochemical reactions that generate cyclobutane pyrimidine dimers (CPDs) and 6-4 pyrimidine-pyrimidone photoproducts. These photoproducts distort the helical structure of DNA and interfere with its replication process. As bacteria cannot multiply, they ultimately die. Although UVC has been used extensively for food disinfection, vacuum sterilization, and other industrial purposes, its application for infection management has only more recently been considered. In low-intensity (1.93 mJ/cm^2^) exposures, as short as 15 s, it has been shown to be effective [103] and safe [104] for managing corneal infection in a murine keratitis model. This differs from corneal collagen crosslinking (CXL), which has demonstrated efficacy in managing bacterial and fungal corneal infections (reviewed in [76]) but uses longer wavelength UV radiation (UVA, 370 nm) in doses as high as 5.40 J/cm^2^ delivered over an extended duration (30–60 min). The dose delivered in CXL is at least 90 times higher than the UVC dose that has been shown to be effective in managing *Pseudomonas* infections. The safety margin with respect to UVC exposure during keratitis treatment is considered high as UVC does not penetrate beyond the first few layers of corneal epithelium, as shown *ex vivo* in porcine eyes and *in vivo* in mouse eyes [103], and is therefore likely to be safe to the corneal stem cells. *In vitro* studies suggest that DNA defects (CPDs) formed after UVC exposure resolve completely within the first few days [103]. In considering possible clinical application, further reassurance is offered by the epithelial cell desquamation process that would see any cells retaining such defects being shed naturally from the cornea and therefore unlikely to carry the defects to subsequent progeny. However, as discussed earlier, even a small dose of UV exposure to the cornea has potential to aggravate viral corneal infections or latent viral disease [79], indicating that more research is required in this area. Further research is thus needed to confirm the potential for UVC technology to be used as an empiric treatment for all types of corneal infections caused by bacteria, fungi, and viruses.

#### 3.1.8. Antimicrobial Blue Light for the Treatment of Microbial Keratitis

As with PDT and UVC, research has described antimicrobial blue light (400–470 nm wavelength) as possessing therapeutic efficacy against a variety of pathogenic microbes present within a variety of localized regions of the body, including the eye [105,106]. Many studies have demonstrated its utility as an adjunctive strategy along with conventional and non-conventional therapeutics [107,108]. The therapeutic potential of antimicrobial blue light in microbial keratitis has been investigated in *ex vivo* and *in vivo* studies [106]. It was found that *Pseudomonas aeruginosa*, a common pathogen associated with MK, could effectively be eliminated within *ex vivo* rabbit corneas and *in vivo* mouse corneas. The *ex vivo* study found that a single antimicrobial blue light exposure (415 nm; 84 J/cm^2^) could reduce the viability of *P. aeruginosa* by 3-log_10_ CFU 6 h post-inoculation of the corneas. However, when corneas were incubated for 24 h, the efficacy was reduced, with an equivalent exposure reducing the effect by less than 1-log_10_ CFU only. Increasing the dose to 304 J/cm^2^ increased the killing to 3-log_10_ CFU, with no apparent regrowth. The *in vivo* study by the same group found that 36 J/cm^2^ was sufficient to reduce the viability of *P. aeruginosa* by 2-log_10_ CFU, 6 h post-inoculation, with lower corneal pathology scores in treated versus untreated mice. In established eye infections (24 h post inoculation), a radiant exposure of 144 J/cm^2^ was sufficient to eradicate *P. aeruginosa* from the corneas. However, the infection recurred in the 6 h and 24 h post-inoculated treatment groups. It was proposed that the concomitant use of antimicrobial blue light plus antibiotic eye drops might limit recurrence, but overall the approach was deemed to have potential value for treating MK caused by drug-resistant organisms. Although antimicrobial blue light demonstrated excellent efficacy in inhibiting bacterial growth in the corneal infection models, its safety to the eye has been a concern as blue light may penetrate deeply, reaching the retina and causing photochemical reactions. Consequently, further studies are warranted in translation of this technology for keratitis management using blue light, with attention paid particularly to the safety issues.

### 3.2. Phage Therapy as an Approach to Combat Microbial Keratitis

Bacteriophages, or phages, are viruses that exclusively infect bacteria. They are like typical viruses in that they cannot replicate autonomously and instead utilize a host (in this case, a bacterium) to propagate their line [109]. They are highly selective in terms of the bacteria they can infect and are found ubiquitously within the environment. The existence of phages has been known for over a century, and their potential to be used therapeutically for treating bacterial infections has been explored since their discovery by Felix d’Herrelle in the early 20th century [110]. Contrary to many novel ‘non-traditional’ therapies, an evidence base exists demonstrating the success of phages in the clinical setting [111]. A number of studies have proposed and investigated the use of phages specifically for the treatment of ophthalmic infections [112,113,114], largely as a last resort when conventional treatments have failed. In one study, phage therapy was investigated for its potential to treat *P. aeruginosa* keratitis [113]. The authors used the *P. aeruginosa*-specific phage, KPP12, prepared and administered as eye drops, for treating MK in a mouse model. It was found that the mock-treated mice developed a severe infection, with a ring abscess being observed on day 1 post-infection, which completely spread throughout the cornea by day 3, and resulted in perforation by day 5. However, the phage-treated mice (treated with a single dose) exhibited only slight opacities at day 1, that almost completely faded by day 5, suggesting that phage therapy significantly improved the clinical outcome and leading to the conclusion that the KPP12 phage might represent a clinically viable alternative therapy for managing *P. aeruginosa* keratitis.

In another study, topical phage therapy was studied in a unilateral corneal infection in a 65-year-old woman with clinical signs of corneal abscess and interstitial keratitis [114]. The patient initially presented with an MRSA infection, requiring the administration of vancomycin, and a corneal transplant was ultimately required due to residual scarring. The patient then incurred a recalcitrant vancomycin-intermediate *S. aureus* infection in the same eye, which proved difficult to treat, with recurrence occurring over a period of eight months. The patient subsequently underwent ‘phage therapy’ to clear the infection and was treated using an *S*. *aureus* phage SATA-8505 for a total of four weeks, administered via topical eye drops and nasal spray, as well as intravenously. The patient was deemed to be clear of infection following assessments at three and six months, leading the authors to conclude that phage eye drops might be a suitable alternative or complementary therapy for MK caused by resistant bacteria. The successful application of ‘phage therapy’ against the corneal infection provided substantial evidence of its potential utility, within the clinical paradigm. It is important to note, however, that further work is needed using other phages targeted against other etiological agents of ocular infection (e.g., *P. aeruginosa* phage, etc.), to evaluate its utility as a broad therapeutic approach. It is conceivable, given the evidence of success of the therapy under clinical conditions, that ‘phage therapy’ may be a suitable approach that may be applied concurrently with conventional antimicrobials, or perhaps as a last resort when the infection resists all forms of traditional treatment.

### 3.3. Probiotics to Eliminate Ocular Pathogens

Human biology operates in concert with many different commensal microorganisms—known as the ‘microbiome’. A suitably diverse local microbiome harboring a variety of commensals is instrumental in permitting the normal functioning of various systems, such as the immune system and digestion [115]. Similarly to the skin, mouth, and gut, the ocular surface also has its own unique but diverse microbiome, composed of culturable and non-culturable microbes that are vital to mitigating eye infections via pathogen inhibition [116]. Therefore, an important question is: can we benefit from the use of probiotics, in the hope of replenishing or revitalizing the ocular microbiome to protect from ophthalmic infections? Several studies have explored the antimicrobial capacity of probiotics in targeting the inhibition of ocular pathogens [117,118,119].

Given the importance of biofilm formation in many infections, including ophthalmic infections, Akova et al. investigated the potential for probiotics to treat biofilm caused by *Bacillus cereus* [117]. They found that biofilm production was lower when testing exopolysaccharides from different species of *Lactobacillus* (lactic acid-forming bacteria) relative to control. Another study sought to investigate probiotics to manage bacterial conjunctivitis. Their study used four species of *Lactobacillus* and two bifidobacterial strains as the relevant probiotics [118]. It was found that when using cell-free preparations of the probiotics, there was significant inhibition of growth by *S. aureus* and *S. epidermidis*, which were also confirmed to be important mediators of bacterial conjunctivitis. All probiotics tested were shown to effectively inhibit both *Staphylococci*. However, *Lactobacillus acidophilus* was found to be the most effective bacterial growth inhibitor. The authors concluded that probiotics may be suitable for treating conjunctivitis secondary to *Staphylococcal* species.

*Neisseria gonorrhoeae* is an important mediator of ophthalmic infection in neonates, causing the condition known as ophthalmia neonatorum. *N. gonorrhoeae* has also been associated with antimicrobial resistance, supporting the need for the development of new treatments [120]. To that end, Ruiz et al. used bacteriocins produced from lactobacilli to inhibit *N. gonorrhoeae in vitro* [119]. It was found that with the use of BLIS-es L23 and L60—the antimicrobial metabolites from lactobacilli—87.2% and 80.66% reductions in viability, respectively, could be achieved. The authors suggested that these lactobacilli-defined metabolites showed potential as possible treatment approaches in combating *N. gonorrhoeae*. It is important to note, however, that the study was not targeted at *N. gonorrhoeae* specifically, as a causative agent of ocular infection, as it was an *in vitro* investigation. It is essential that further studies are carried out, *in vivo*, to determine how these metabolites interact with the ocular microbiome, and how this impacts the progression of ophthalmia neonatorum.

## 4. Treatment of Ophthalmic Infections: Can We Shift the Paradigm?

This review discussed ophthalmic infections, their etiologies, current treatments, and antimicrobial resistance as a compromiser of therapeutic efficacy. Also, potential non-traditional methods to overcome antimicrobial resistance and facilitate the timely treatment of ophthalmic infections were summarized. However, it is important to recognize the limitations of potential novel technologies, which require further exploration through well-designed research questions and methods.

PDT has shown significant potential as a treatment for a variety of extraocular infections. It shows benefits as a rapid, non-selective, and localized treatment approach, where systemic treatment can fail to reach an adequate therapeutic level for management of localized infection [121]. However, as with all therapeutic strategies, PDT has several limitations. As it relies on the combination of light and a photosensitizer to manage infection, it is essential that both components reach the site of infection. PDT treatment of corneal eye infections appears imminently practical given that topical application of the photosensitizer, coupled with light (wavelengths ranging from 350 nm to 660 nm), reaches the deeper layers of the cornea with minimal attenuation. Concurrent damage to host cells is likely as the ROS are not selective only to microbes. Only a limited number of studies have explored the therapeutic benefits of this technology *in vivo*, highlighting a gap in research translation. Except for CXL technology, only a few PDT studies have been tested clinically, which raises the question of therapeutic validity.

UVC is intrinsically antimicrobial as it is absorbed by nucleic acids resulting in a myriad of destructive effects that effectively inactivate pathogens. It can kill microorganisms irrespective of their genetic and phenotypic makeup and has been proven effective against bacteria, fungi, and viruses. Recent studies have made successful efforts to characterize the safety and efficacy of UVC to manage corneal infections, bringing this technology a step closer to clinical translation.

Antimicrobial blue light is similar to PDT in that it induces a photodynamic reaction but does not require an exogenous photosensitizer as is required in PDT. It harnesses intrinsic chromophores present within microbes to produce antimicrobial effects. It may prove to be a practical solution to managing localized infection; however, concerns regarding its uses in ophthalmic infection management remain. Blue light may lead to the development of cataracts [122] and can reach the retina and cause photochemical reactions leading to safety concerns [122]. However, it is important to note that, as with all therapeutic interventions, a ‘therapeutic window’ may exist whereby lower antimicrobial blue light exposures may be safe [123], but further work is required to substantiate this idea. The effective and rapid elimination of pathogens with aBL, along with its validation *in vivo,* and cost-effectiveness, suggests that aBL has potential to develop into a useful approach to managing corneal infections if the inherent limitations can be overcome.

Recently, phage therapy has experienced a surge in interest as a last-resort therapeutic technique for treating highly resistant bacterial infections. It is highly selective towards specific pathogens, which may limit its impact on the ocular microbiome. Furthermore, its extensive clinical validation against infections (including eye infections) proves its feasibility for infection management. However, a potential limitation is that phage therapy of ocular infections has a narrow spectrum of activity as it is bacterium-specific (e.g., *P. aeruginosa* phages cannot eliminate *S. aureus*) [123]. This specificity indicates that the etiology of infection must be fully understood before applying phage therapy. Phage therapy may not prove ideal for the management of corneal infections, where delayed diagnosis in identifying causative pathogens can lead to rapid progression of ocular infection. A significant amount of work is still needed to characterize the different phages [124] and identify rapid methods towards their preparation, limiting their usefulness at the current time.

Probiotics have also been investigated therapeutically for ocular infections. These have the benefit of replenishing the ocular microbiome, an important strategy to protect against invasion by pathogenic microbes. Growing evidence regarding the validity and safety of probiotics in several clinical paradigms [125] lends support for such a strategy to prevent or manage ocular infections. However, there is still a need for more substantive evidence supporting their clinical application to validate their effectiveness as a treatment for eye infections. Furthermore, there is a gap in the literature as to how the different probiotics might interact with all the different etiological agents of infection, limiting the ability to assess their effectiveness in managing deeper ocular infections.

## 5. Conclusions

A surge in corneal infections is being witnessed as a result of antibiotic-resistant bacteria, with increasing evidence of unsuccessful treatment outcomes from established therapies. Commonly used ophthalmic antibiotics that previously were highly effective in treating eye infections are showing an increasing loss of efficacy in managing infection due to bacteria, fungi, viruses, and Acanthamoeba. Without the development of new effective pharmacologically based anti-infective agents, alternative methods of targeting infections are needed. Several novel methods are under investigation, including light-based anti-infective technology with or without chemical substrates, phage therapy, and probiotics. Multiple preclinical studies and a limited number of clinical case studies have confirmed the efficacy of some of these novel methods. However, given the fast pace at which corneal infections evolve, any treatment requires immediate institution for a rapid effect to prevent complications such as loss of vision and corneal perforation. Given their rapid effects on microbial viability, light-based technologies seem particularly promising in this regard. However, with respect to more severe or established corneal infections that have become recalcitrant, the application of phage therapy may be a useful adjunct or replacement therapy to provide a sustained curative treatment.

## Data Availability

Not applicable.
